# Double expansible ring annuloplasty for a dynamic Ross procedure

**DOI:** 10.1016/j.xjtc.2025.09.018

**Published:** 2025-10-06

**Authors:** Claudia Côté, Luigi Garufi, Pavel Zacek, Margaux Bernardini, Pascal Leprince, Emmanuel Lansac, Pichoy Danial

**Affiliations:** a Division of Cardiac Surgery, Department of Surgery, Halifax Infirmary, Dalhousie University, Halifax, Nova Scotia, Canada; b Department of Cardiovascular and Thoracic Surgery, Sorbonne University, Institute of Cardiology, Pitié-Salpêtrière Hospital, Assistance Publique-Hôpitaux de Paris, Paris, France; c Department of Cardiac Surgery, Charles University, Faculty of Medicine in Hradec Kralove and University Hospital in Hradec, Kralove, Czechia

**Figure:**
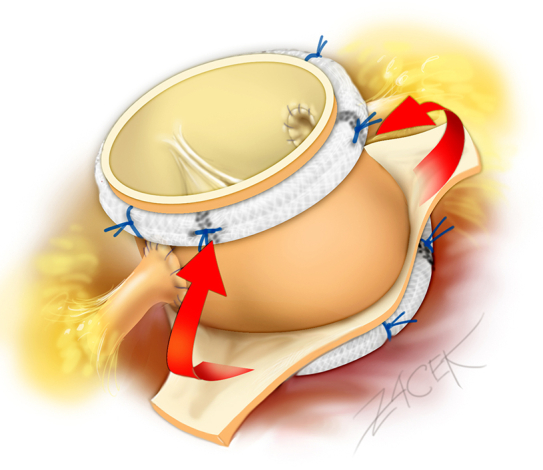
Double ring annuloplasty for a dynamic Ross procedure.

**Central Message**

The Ross procedure with external double expansible ring annuloplasty restores a dynamic ratio between the annulus and STJ to prevent recurrence of aortic dilatation and regurgitation.

The Ross procedure offers better long-term survival and freedom from valve-related complications compared with prosthetic aortic valve replacement.^1^ However, progressive autograft dilatation with concurrent regurgitation occurs in 10% to 30% of patients, particularly when aortic regurgitation is the primary indication and with annular dilation (>25 mm).^2^ Techniques to avoid this complication, such as inclusion within a graft, have been described. Recently, a more physiological approach was proposed with an extra-aortic annuloplasty and a short ascending aortic tube graft replacement as well as inclusion within the remnant root tissue (ie, noncoronary sinus and right-left commissure).^3^^,^^4^ In the absence of ascending aortic dilatation, we propose a modification to restore the sinotubular junction (STJ) and annulus ratio with a double external subannular and STJ expansible ring annuloplasty. This study was approved by the Aortic Valve Insufficiency and ascending aorta Aneurysm InternATiOnal Registry review boards (NCT00478803; May 25, 2007). Individual patient consent was waived.

## Technique

The procedure was performed via median sternotomy on cardiopulmonary bypass using standard techniques. The pulmonary trunk was opened and the pulmonary valve was inspected. The pulmonary valve annulus measured 25 mm. The diseased aortic valve was excised, coronary buttons were prepared, and the aortic root was dissected as far as the level of the aortic annulus (Video 1). The diameter of the native aortic annulus was measured using Hegar dilators and the size of the annuloplasty rings used was based on the size of the Hegar dilator (Table 1) aiming to upsize the ring by approximately 2 sizes (2 mm) relative to the Hegar measurement, ensuring an annuloplasty that avoids stenosis. The aortic valve annulus measured 27 mm. Pledgeted 2-0 polyester braided mattress sutures were placed circumferentially, from inside out in the subvalvular position, at the nadirs of each cusp insertion and at the base of the interleaflet triangles between the right/left and non/left coronary sinus. The sixth pledgeted suture was added externally at the level of the right/noncoronary commissure to avoid potential injuries to the bundle of His. The pulmonary autograft was harvested.

**Table 1:** Criteria for choice of the subvalvular aortic ring and sinotubular ring size

Variable	Surgical aortic annular size (Hegar) (mm)		
	25-27	28-30	≥31-31
Subvalvular extra-aortic ring (mm)	29	31	33
Sinotubular junction extra-aortic ring	25-27∗	27-29∗	29-31∗

∗ Depending on size of neosinotubular junction on pulmonary autograft.

**Figure 1:**
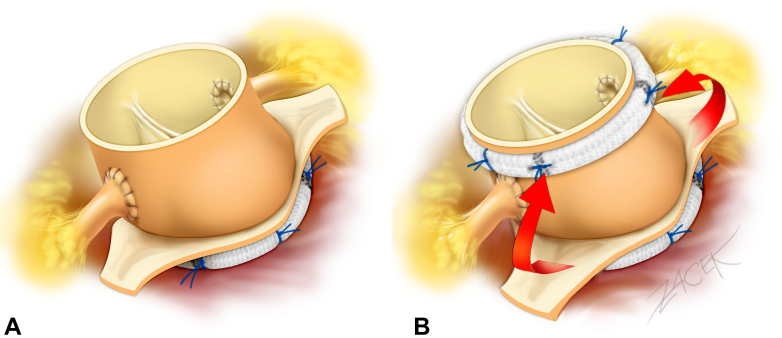
A, Pulmonary autograft with subannular ring in situ. B, Pulmonary autograft with double annuloplasty. *Arrows* indicate position of native aortic remnant to be tacked to extra-aortic ring.

The subvalvular stitches were then passed through a 29-mm extra-aortic ring (Coroneo Inc) and tied down externally at the level of the aortic annulus. The proximal anastomosis of the autograft was performed using interrupted 2-0 nonpledgeted polyester braided sutures. Coronary buttons were reimplanted (Figure 1). Five 2-0 pledgeted polyester braided mattress sutures were placed around the STJ: 1 at each commissure and 1 above each coronary. A 27-mm extra-aortic ring (Coroneo Inc) was implanted at the STJ. The remaining native aortic tissue of the noncoronary sinus and the left-right commissure are tacked to the ring to provide external, nonrestrictive support to the autograft root (Figure 1). Finally, the continuity of the right ventricular outflow tract was restored with a cryopreserved pulmonary homograft. Postoperative echocardiography scans showed no regurgitation and a mean transvalvular gradient of 3 mm Hg.

## Discussion

This is a sound modification to the Ross procedure that can be applied to all adult-sized patients. Polyethylene terephthalate graft reinforcement prevents long-term dilation but at the expense of aortic root dynamics. The extra-aortic ring provides 10% expansibility with stable results over time.^5^ The proposed use of an extra-aortic ring is simple and reproducible, with no need to reimplant the commissures within a graft.^6^ Instead of replacing a nondilated ascending aorta to stabilize the STJ in the autograft, the use of an STJ ring avoids the need for a polyethylene terephthalate tube that maintains aortic compliance, eliminates the distal anastomosis, and preserves endocardial continuity in the root and ascending aorta preserving endothelial function.

This report is limited by being a single case. This technique has been used in 5 patients at our institution, and we plan to use this technique routinely going forward. Long-term follow-up will be captured as part of the Heart Valve Society prospective database.

## Conclusions

The Ross procedure with external double expansible ring annuloplasty restores a dynamic ratio between the annulus and STJ to prevent recurrence of aortic dilatation and regurgitation.

## Conflict of Interest Statement

Dr Lansac has consultant agreements with Coroneo Inc and has received patents and royalties from Extra-Aortic Ring (Coroneo Inc). All other authors reported no conflicts of interest.

The *Journal* policy requires editors and reviewers to disclose conflicts of interest and to decline handling or reviewing manuscripts for which they may have a conflict of interset. The editors and reviewers of this article have no conflicts of interest.

## Supplementary Data

Video 1 Operative technique of the Ross procedure with double annuloplasty. Video available at: https://www.jtcvs.org/article/S2666-2507(25)00437-7/fulltext.
