# Supplementary figures and images for: Double expansible ring annuloplasty for a dynamic Ross procedure
Source: JTCVS Tech. 2025 Oct 6;34:67–9. doi: 10.1016/j.xjtc.2025.09.018 (PMC12683052; doi:10.1016/j.xjtc.2025.09.018)

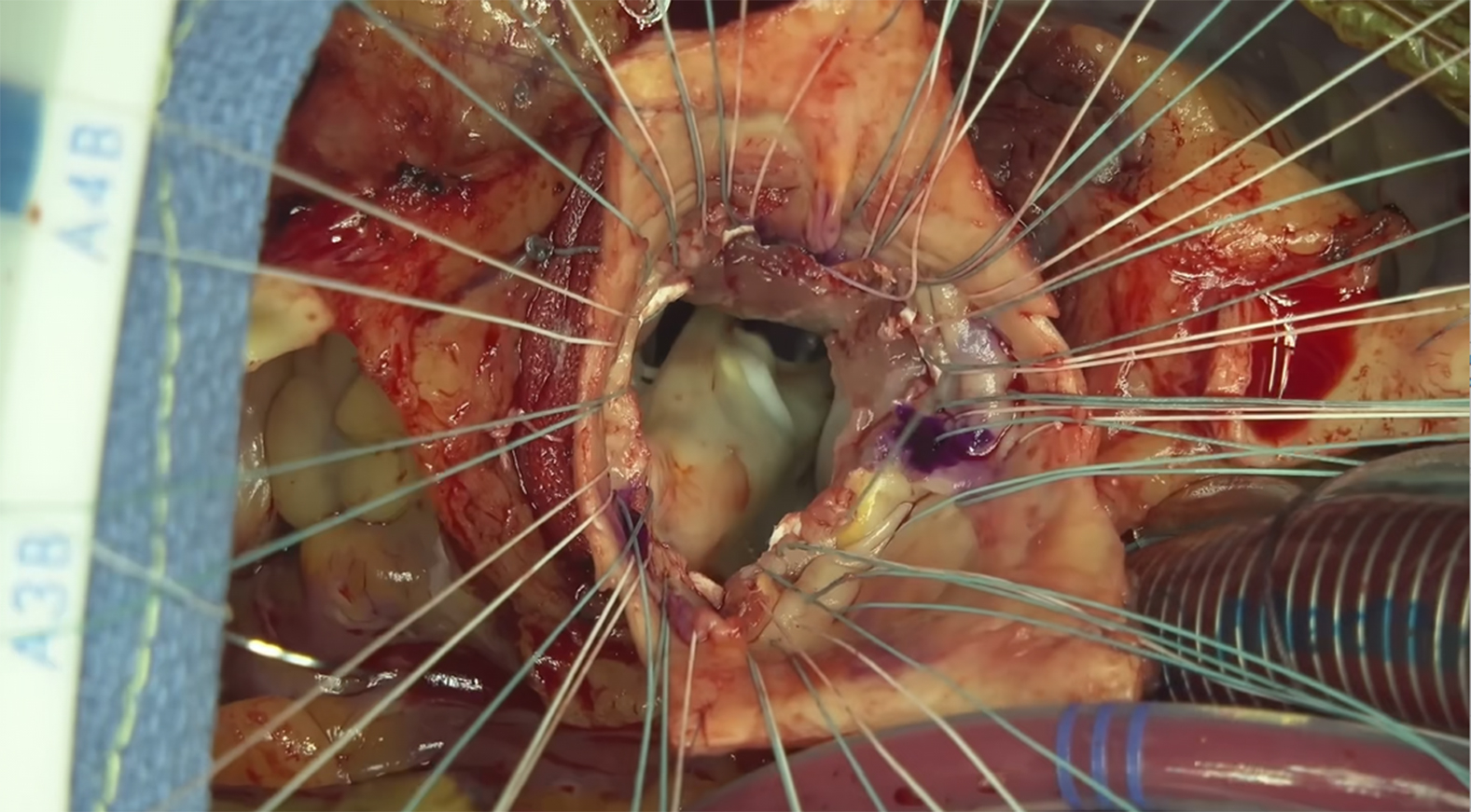

Supplement: Video 1 — Operative technique of the Ross procedure with double annuloplasty. Video available at: https://www.jtcvs.org/article/S2666-2507(25)00437-7/fulltext. [file fx2.jpg]
